# ATF4 overexpression induces early onset of hyperlipidaemia and hepatic steatosis and enhances adipogenesis in zebrafish

**DOI:** 10.1038/s41598-017-16587-9

**Published:** 2017-11-27

**Authors:** Kun-Yun Yeh, Chi-Yu Lai, Chiu-Ya Lin, Chia-Chun Hsu, Chung-Ping Lo, Guor Mour Her

**Affiliations:** 1 Division of Hemato-Oncology, Department of Internal Medicine, Chang-Chung Memorial Hospital, 222 Maijin Road, Keelung, 204 Taiwan; 2 Department of Bioscience and Biotechnology, National Taiwan Ocean University, 2, Pei Ning Road, Keelung, 202 Taiwan; 3 Department of Radiology, Buddhist Tzu Chi General Hospital, Taichung Branch, No. 66 Fēngxìng Road Section 1, Taichung, 427 Taiwan; 4 School of Medicine, Tzu Chi University, No.701, Sec. 3, Jhongyang Road, Hualien, 97004 Taiwan

## Abstract

Activating transcription factor 4 (ATF4) is constitutively expressed in a variety of tissues, and regulates several pathological features associated with metabolic diseases such as non-alcoholic fatty liver diseases (NAFLD) and obesity. However, the role of ATF4 in animal model systems is poorly understood. To investigate ATF4 functions in zebrafish, we conditionally expressed ATF4 proteins, using a Tet-off transgenic system. We observed early-onset hyperlipidaemia and liver steatosis in ATF4 transgenic zebrafish (ATs) without doxycycline treatment (ATs − Dox). Oil Red O (ORO)-stained signals were predominant in the intravascular blood vessels and liver buds of larval ATs − Dox, indicating that ATF4 functionally promotes lipogenesis. Further, ATF4 overexpression accompanied the stimulation of the unfolded protein response. Therefore, adult ATs − Dox showed increased lipid accumulation, which led, in turn, to liver steatosis. Liver histology and ORO staining of ATs − Dox hepatocytes also indicated oxidative stress and induced NASH-like phenotypes. Moreover, ATF4 overexpression accelerated adipocyte differentiation via CCAAT enhancer binding protein-beta and peroxisome proliferator activated receptor-gamma inducible expression. ATs-Dox zebrafish showed increased weight gain with larger fat pads due to adipocyte hyperplasia. In this study, we report that ATF4 is a potential stimulator of lipid biosynthesis and adipogenesis in zebrafish.

## Introduction

Activating transcription factor 4 (ATF4), also known as cAMP-response-element-binding protein 2 (CREB2), Tax-Responsive Enhancer Element B67 (TAXREB67) or CCAAT/enhancer binding proteins related activating transcription factor (C/ATF)^1–4^, belongs to the family of basic zipper-containing proteins. ATF4 is constitutively expressed in a wide variety of tissues, including brain, heart, white adipose tissue (WAT), brown adipose tissue (BAT), liver, spleen, thymus, lung, and kidney^3,5^. It has been reported that ATF4 is involved in the regulation of various biological processes, including long-term memory^6^, osteoblast differentiation^7^, endoplasmic reticulum (ER) stress^8,9^, amino acid deprivation^10^, glucose metabolism^11^, redox homoeostasis^12^, and lipid metabolism and thermogenesis^13^.

ATF4 deficiency protects mice from high-carbohydrate diet-induced liver steatosis^14–16^. Seo *et al*. observed that ATF4-null mice do not develop non-alcoholic fatty liver diseases (NAFLD) when induced by a high-fat diet (HFD)^11^. Wang *et al*. observed that knockdown of ATF4 in the liver protects against HFD-induced oxidative stress and triglyceride (TG) accumulation both *in vitro* and *in vivo* ^17^. However, Xiao *et al*. showed that ATF4 gain-of-function was associated with increased TG synthesis and secretion secondary to augmented lipogenesis in primary hepatocytes of C57BL/6 J mice^16^. In addition, ATF4 has been shown to play an important role in the regulation of adipocyte differentiation^18^. ATF4 determines adipocyte differentiation, by regulating CCAAT enhancer binding protein-beta (C/EBP-β) and peroxisome proliferator activated receptor-gamma (PPAR-γ)^18,19^. Overexpression of ATF4 accelerates adipocyte differentiation, whereas knockdown of ATF4 inhibits adipocyte differentiation and adipogenic gene expression^18^.

The unfolded protein response (UPR) allows cells to adapt to changing physiological demands and extreme stress by enhancing protein folding and quality control in the ER and modulating the influx of nascent proteins into the ER. Fatty liver disease (FLD) is associated with markers of UPR activation and robust UPR induction can cause steatosis^20^. The UPR is activated in obesity-associated FLD and alcohol-induced liver injury, which are concomitant with steatosis, thus raising the possibility that ER stress-dependent alteration in lipid homoeostasis is the mechanism that underlies this steatosis^21^. It has been shown that ATF4 is a transcription factor representing one of three branches of the UPR that is activated in cells in response to ER stress^22–24^. It has been hypothesized that adipocytes elevate the UPR to alleviate ER stress caused by adipogenesis^25^. Some UPR regulators have been reported to be required in adipocyte differentiation^19,26,27^. Furthermore, ATF4 also serves as a master regulator of the UPR that activates the induction of downstream UPR genes^28^, and has been demonstrated to function as a novel positive regulator of the differentiation of preadipocytes into adipocytes through the induction of C/EBP-β and PPAR-γ expression^18^.

The zebrafish is emerging as a useful animal model for studying lipid metabolism and mechanisms of human disease related to lipid abnormalities^29–33^. The aim of the present study was to explore the effects of ATF4 and expression of its target genes on the lipid metabolism of both larval and adult zebrafish. We generated ATF4 transgenic zebrafish to analyse the roles of the zebrafish liver ATF4 protein during lipid homeostasis. We report that ATF4 overexpression induces intravascular lipid accumulation and enhances adipogenesis in zebrafish via the UPR.

## Results

### Generation of transgenic zebrafish with conditional expression of ATF4

**Figure 1:**
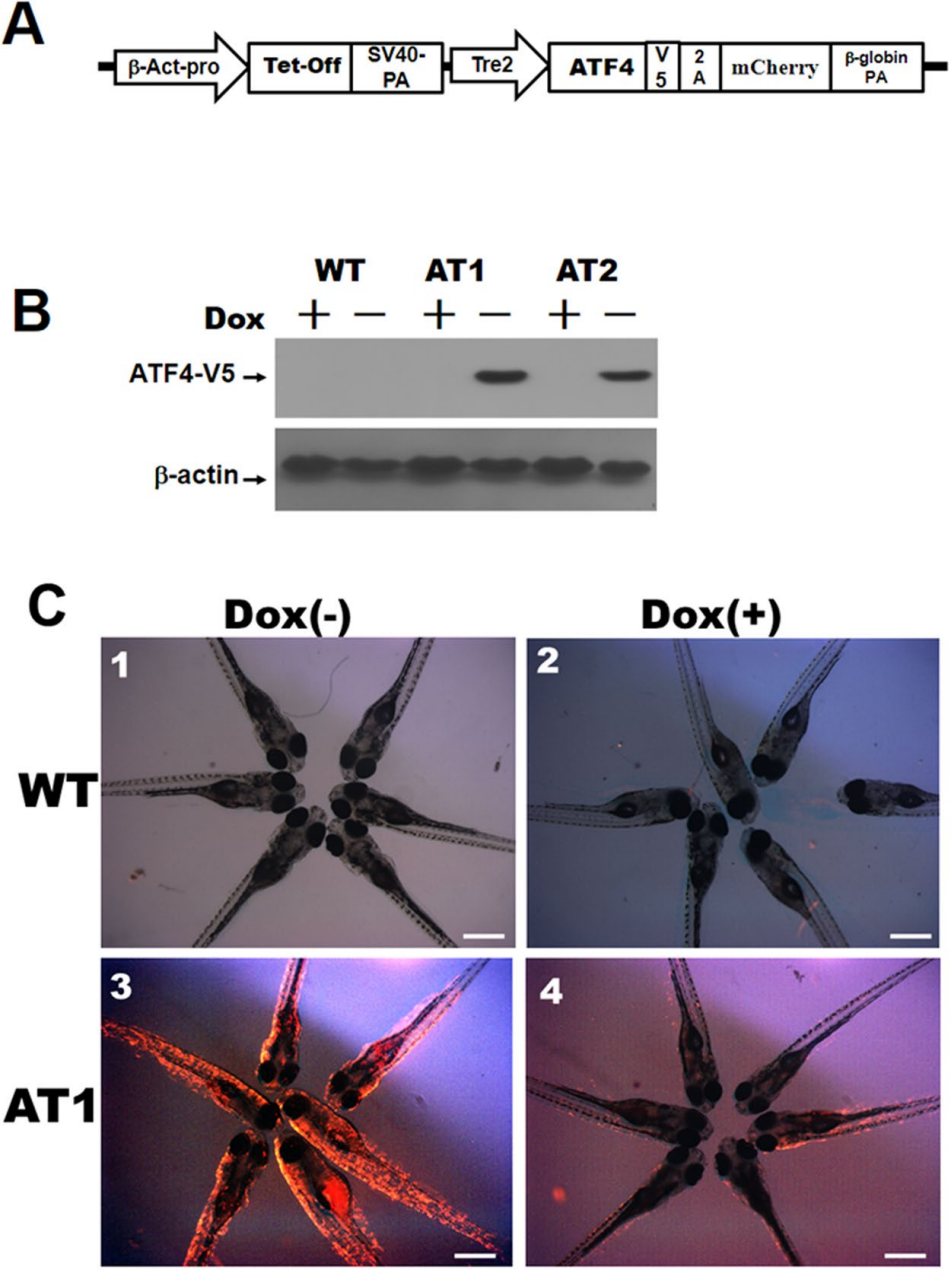
Generation and characterization of zebrafish transgenic ATF4 lines (ATs). (**A**) The transgenic construct, pβ-Act-Tet^off^-ATF4-2A-mCherry. (**B**) Immunoblot analysis of ATF4 expression in AT1 and AT2 fish. Control: wild type (WT). (**C**) AT1 and WT without Dox treatments (−Dox, panels 1 and 3, 40X magnification, scale bars: 200 μm), and AT1 and WT with (+Dox, panels 2 and 4, 40X magnification, scale bars: 200 μm).

We observed various levels of endogenous ATF4 mRNA expression in various tissues of adult zebrafish, including the liver, intestine, brain, muscle, adipose tissue, and eye (Supplementary Fig. S1). To induce stable conditional expression of the *ATF4* gene in zebrafish, we used the pβ-Act-Tet^off^-ATF4–2A-mCherry construct (Fig. 1A) to produce germline-transmitting transgenic zebrafish lines. Two stable ATs, [Tg (−2.5β-Act:Tet^off^-ATF4–2A-mCherry)], were designated as AT1 and AT2. The ATs all expresses high levels of the ATF4 protein without doxycycline treatment (−Dox), whereas no ATF4 protein was detected in Dox treatment groups (+Dox). The ATs showed no discernible phenotypic changes in two founder lines at early embryonic stages compare to WT (wild type) (data not shown); the AT1 line expressed slightly higher levels of the ATF4 protein, thus, the majority of the results given in this study are for AT1 zebrafish (Fig. 1B). At 6 dpf, the size of AT1 ± Dox larvae was the same as that of the WT ± Dox larvae (Fig. 1C). No mCherry fluorescence was observed in WT ± Dox larval samples (Fig. 1C, panels 1 and 2). However, the strong mCherry fluorescence in AT1 − Dox larvae (Fig. 1C, panel 3) comprises the weak to no or less mCherry fluorescence seen in AT1 + Dox larvae (Fig. 1C, panel 4).

### ATF4 overexpression increases endotrophic and intravascular lipid accumulation in larval ATs

**Figure 2:**
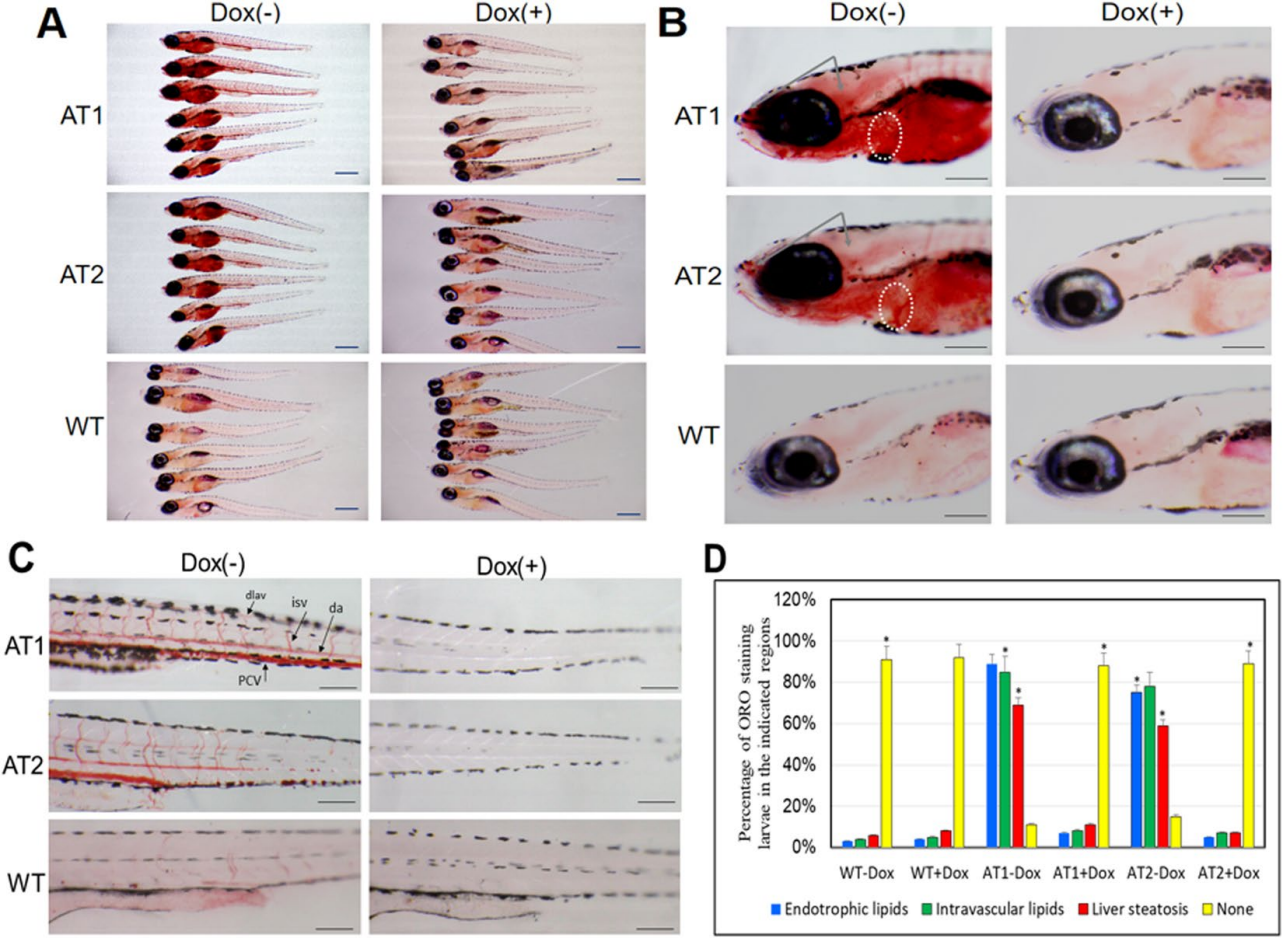
Induction of endotrophic and intravascular lipid accumulation in larval ATs. (**A**) Whole-mount ORO staining of 9-days post fertilization (dpf) ATs ± Dox and WT ± Dox larvae (32X magnification, scale bars: 200 μm). (**B**) Enlargement at the brain region in (A) shown lipid accumulation in brain and heart. ORO stained brain regions are indicated by the arrows. Hearts are circled (110X magnification, scale bars: 200 μm). (**C**) Enlargement at the posterior level in (A) shown intravascular lipid accumulation (110X magnification, scale bars: 200 μm). (**D**) Cumulative percentage of ORO staining zebrafish larvae in the indicated regions. Statistical analyses of ATs ± Dox and WT ± Dox larvae with liver steatosis and intravascular lipids was calculated through ORO staining at 9 dpf. The ORO staining were performed in triplicate with on average 60–80 larvae per groups (WT-Dox, WT + Dox, AT1-Dox, AT1 + Dox, AT2-Dox, and AT1 + Dox). The asterisk represents statistically significant differences; *p < 0.01, and **p < 0.005. Abbreviations: da, dorsal aorta; dlav, dorsal longitudinal anastomotic vessel; isv, intersegmental vessel; pcv, posterior cardinal vein.

To study the functional role of ATF4 in zebrafish lipid metabolism, we examined the lipid content in larval ATs. To examine the lipid contents among larval ATs, at 9 days post fertilization (dpf), larvae were stained with Oil Red O (ORO) (Fig. 2). ORO signal for endotrophic lipids was detected in 9-dpf ATs − Dox fish (Fig. 2A), while endotrophic lipid levels of ATs + Dox and WT ± Dox fish were not significantly detected; ORO stained the swim bladder non-specifically. In ATs − Dox stained larvae, strong ORO staining also appeared in the liver with additional staining that extended to the brain and heart (Fig. 2B) compared to that observed in the ATs + Dox and WT ± Dox stained larvae. Most interestingly, the ATs − Dox larvae also showed strong ORO staining in the whole vasculature, including the posterior cardinal vein, dorsal aorta, and intersegmental vessels (Fig. 2C), whereas the ATs + Dox and WT ± Dox stained larvae showed mild ORO staining in the anterior intestine region (Fig. 2B). Importantly, the incidence of endotrophic, intravascular, or liver lipid accumulation was much higher in the AT1 − Dox (69–89%) and AT2 − Dox (59–78%) fish compared with only 5–11% for ATs + Dox larvae or 3–8% for WT ± Dox larvae (Fig. 2D). These data suggest that ATF4 overexpression can induce endotrophic and intravascular lipid accumulation in larval zebrafish.

### ATF4 overexpression increases gene expression associated with lipogenesis and the UPR

**Figure 3:**
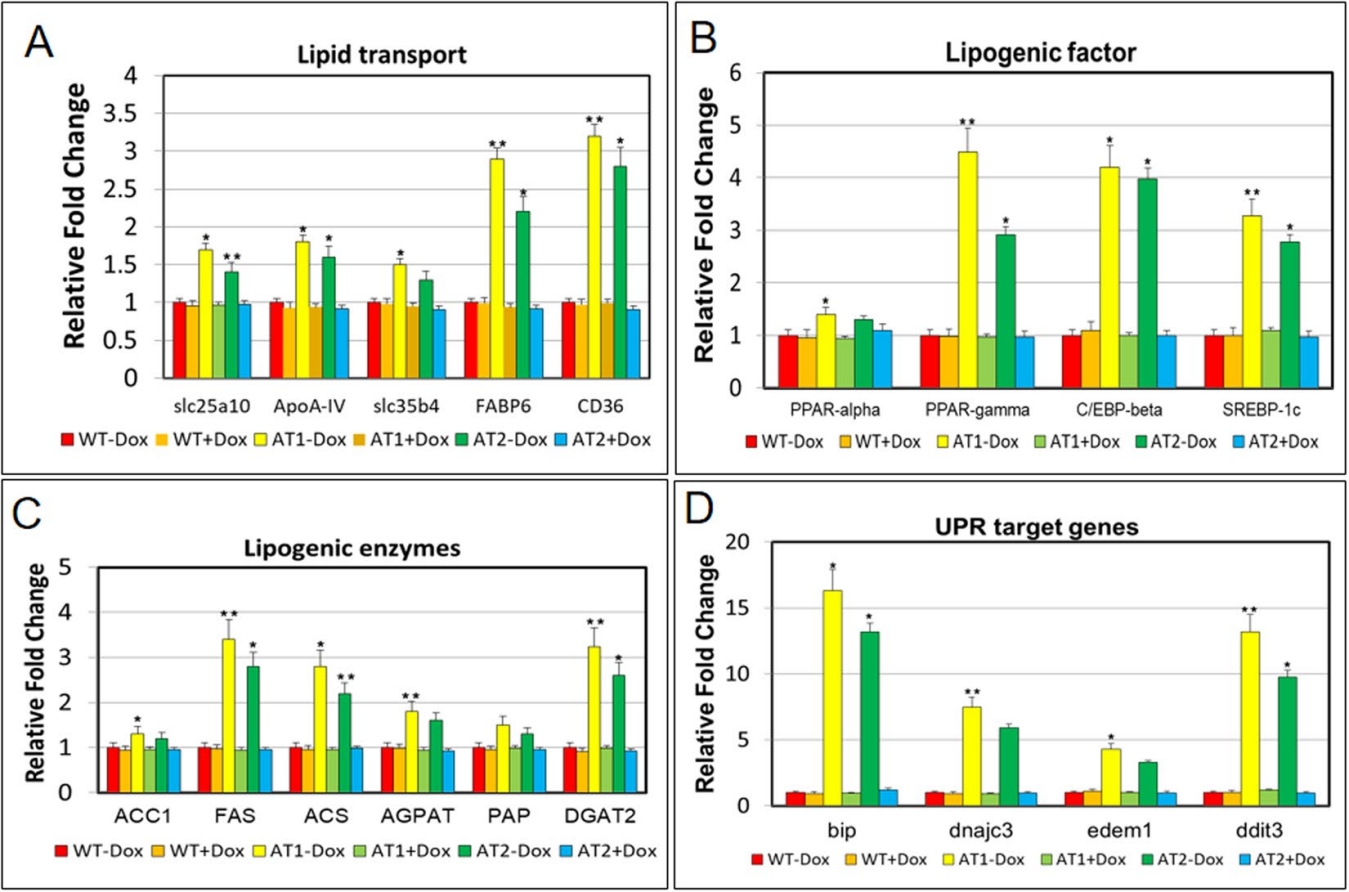
Quantitative real-time polymerase chain reaction (qRT-PCR) analysis of selected lipogenic and UPR target genes in ATs. The relative mRNA expression of lipid transport (**A**), lipogenic factor (**B**), lipogenic enzyme (**C**), and UPR target (**D**) genes in ATs − Dox compared with gene expression in controls (ATs + Dox and WT ± Dox). The qRT-PCRs were performed in triplicate. Expression analysis of the selected genes using cDNA prepared from average 2–3 four months male–female fish pairs per groups (WT-Dox, WT + Dox, AT1-Dox, AT1 + Dox, AT2-Dox, and AT2 + Dox). Levels of mRNA were normalized to β-actin and expressed as fold of values in the WT-Dox control. Values were normalized against β-actin. The asterisk represents statistically significant differences; *p < 0.01, and **p < 0.005.

Since ATF4 overexpression can induce endotrophic and intravascular lipid accumulation, and hepatic steatosis in zebrafish, we then investigated the effect of ATF4 overexpression on the expression of lipogenic and UPR target genes. The mRNA levels of the fatty acid (FA) membrane transporters exhibited significant upregulation, including that of fatty acid transport proteins (FATPs) such as slc25a10 and slc35b4, fatty acid binding proteins (FABPs) such as FABP6, and fatty acid translocase (FAT)/CD36 in ATs − Dox fish compared with the expression observed in ATs + Dox and WT ± Dox fish (Fig. 3A). Expression of genes involved in hepatic lipogenesis that are transcriptionally regulated by sterol regulatory element binding transcription factor 1c (SREBP-1c), including PPAR-γ and C/EBP-β, was also increased (Fig. 3B). The mRNA levels of key lipogenic enzymes involved in fatty acid synthesis were also significantly increased, including acetyl-CoA carboxylase 1 (*ACC1*), fatty acid synthase (FAS), acyl-CoA synthase (*ACS*), acyl-CoA:1-acylglycerol-sn-3-phosphate acyltransferase (*AGAPT*), phosphatidic acid phosphatase (*PAP*), and diacylglycerol O-acyltransferase 2 (*DGAT2*) (Fig. 3C). Additionally, expression of four UPR target genes were also significantly increased, including, *bip*, *dnajc3*, *edem1*, and *ddit3* (Fig. 3D). All markers were significantly and highly upregulated in AT − Dox fish compared with that in the controls (AT + Dox and WT ± Dox). These results suggest that lipid accumulation in the ATs results from upregulated genes involved in lipid biogenesis and UPR activation.

### ATs − Dox fish develop different levels of hepatic steatosis

**Figure 4:**
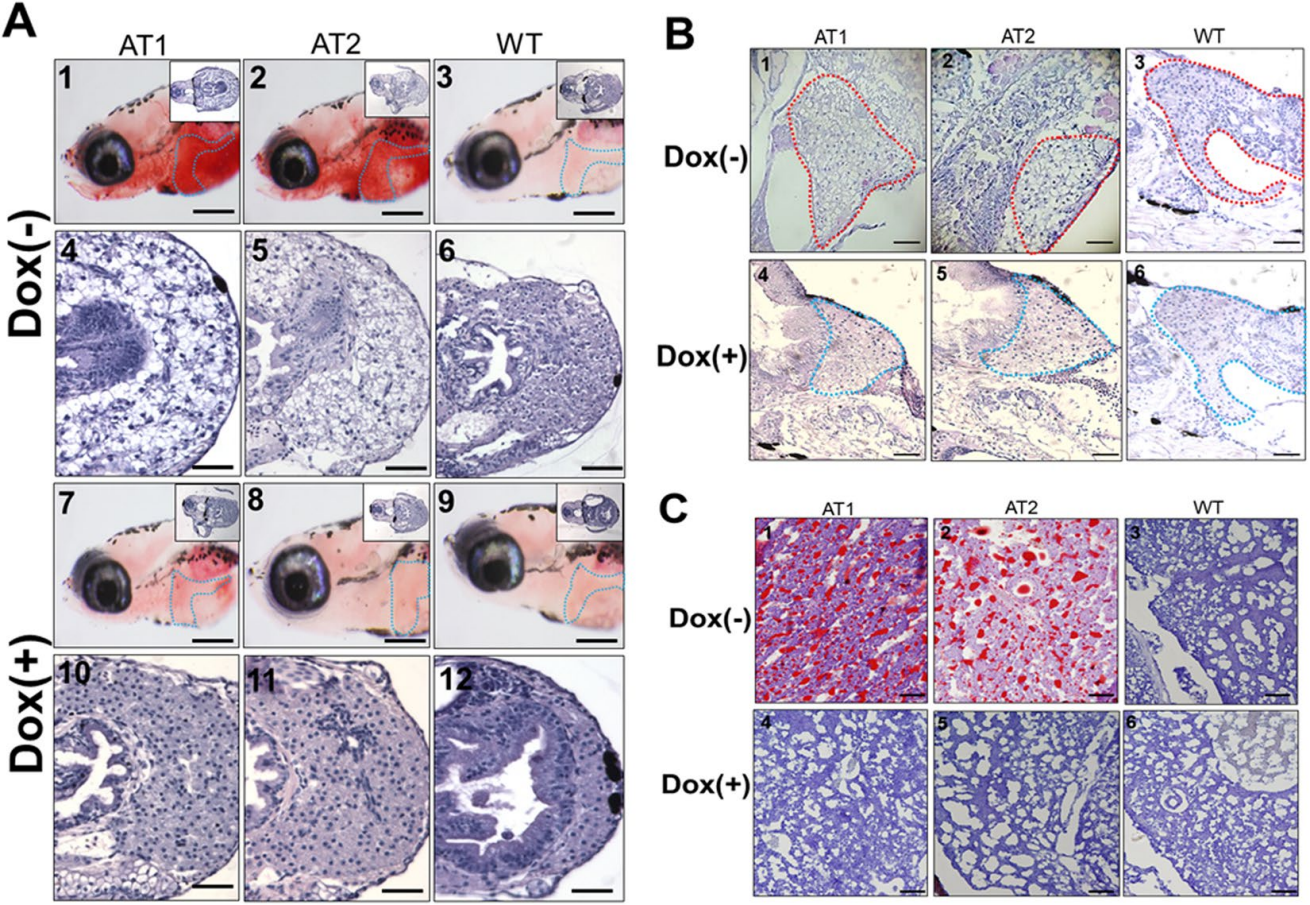
Histological changes in livers of ATs − Dox larvae and juvenile ATs (<30 days post fertilization (dpf)) at different quantities of feeding and lipid contents. (**A**) Whole-mount ORO staining in the liver region of AT1 ± Dox (panels 1 and 7), AT2 ± Dox (panels 2 and 8), and WT ± Dox (panels 3 and 9) larvae at 10 dpf (110X magnification, scale bars: 100 μm). Livers are circled. H&E staining in the liver of AT1 ± Dox (panels 4 and 10), AT2 ± Dox (panels 5 and 11), and WT ± Dox (panels 6 and 12) larvae. (400X magnification, scale bars: 200 μm) (**B**) H&E staining in the liver region of juvenile (28 dpf) AT1 ± Dox (panels 1 and 4), AT2 ± Dox (panels 2 and 5), and WT ± Dox (panels 3 and 6). Livers are circled. 200X magnification, scale bars: 200 μm (**C**) Frozen section ORO staining (frozen ORO) in the liver of juvenile (28-dpf) AT1 ± Dox (panels 1 and 4), AT2 ± Dox (panels 2 and 5), and WT ± Dox (panels 3 and 6). 400X magnification. Scale bars: 10 μm.

Whole-mount ORO staining showed lipid accumulation in the embryonic livers of ATs–Dox fish (Fig. 2B). Thus, we suspected that the early onset of NAFLD phenotypes developed in larval ATs − Dox. As expected, larval ATs − Dox at 14 dpf developed obvious liver steatosis (Fig. 4A, panels 1 and 2), whereas AT + Dox (Fig. 4A, panels 7 and 8) and WT ± Dox larvae (Fig. 4A, panels 3 and 9) given the same diet displayed no or few lipid deposits. A histological analysis indicated that ATs − Dox liver cells underwent a ballooning degeneration process (Fig. 4A, panels 4 and 5) compared to that the liver cells in the ATs + Dox (Fig. 4A, panels 10 and 11) and WT ± Dox (Fig. 4A, panels 6 and 12) groups.

We then examined liver pathogenesis in juvenile ATs + Dox (28 dpf). Histological analysis revealed that liver sections of ATs − Dox juveniles exhibited hepatic cells with various grades of vacuolization in the cytoplasm (Fig. 4B, panels 1 and 2). However, homogeneous liver sinusoids and hepatic cells exhibited a well-preserved cytoplasm and prominent nucleus in ATs + Dox juveniles (Fig. 4B, panels 4 and 5) and WT ± Dox (Fig. 4B, panels 3 and 6). H&E staining results were further confirmed using ORO staining, which clearly revealed vesicular steatosis and massive lipid droplets in the liver tissues from ATs − Dox juveniles (Fig. 4C, panels 1 and 2) compared with those in juvenile ATs + Dox (Fig. 4C, panels 4 and 5) and WT ± Dox (Fig. 4C, panels 3 and 6). Thus, the livers of ATs-Dox juveniles exhibited significantly developed NAFLD compared with that of the controls. Furthermore, the TG content in both male and female livers of ATs − Dox adults was significantly increased compared with that in livers of the ATs + Dox and WT ± Dox adults (Table 1). These results suggest that ATF4 overexpression induces different grades of hepatic steatosis in the zebrafish liver.

**Table 1:** Comparison of liver triglyceride content in both male and female livers of ATs and WT adults with Doxycycline (Dox) treatment.

Lines	Sex	Doxycycline (Dox)	Liver Triglyceride (μmol/g)
AT1	Male	−Dox	50.31 ± 0.45
AT1	Female	−Dox	55.86 ± 0.27^*^
AT1	Male	+Dox	23.19 ± 0.44
AT1	Female	+Dox	25.67 ± 0.42^*^
AT2	Male	−Dox	45.11 ± 0.25^*^
AT2	Female	−Dox	48.26 ± 0.71^*^
AT2	Male	+Dox	22.41 ± 0.15
AT2	Female	+Dox	21.37 ± 0.22^*^
WT	Male	−Dox	18.21 ± 0.35
WT	Female	−Dox	20.56 ± 0.17^*^
WT	Male	+Dox	20.45 ± 0.51
WT	Female	+Dox	22.87 ± 0.42^*^

^*^Significant difference between WT and ATs (*p* < 0.05).

### ATF4 overexpression enhances zebrafish from high fat diet (HFD) induced oxidative stress and induces NASH-like phenotypes

**Figure 5:**
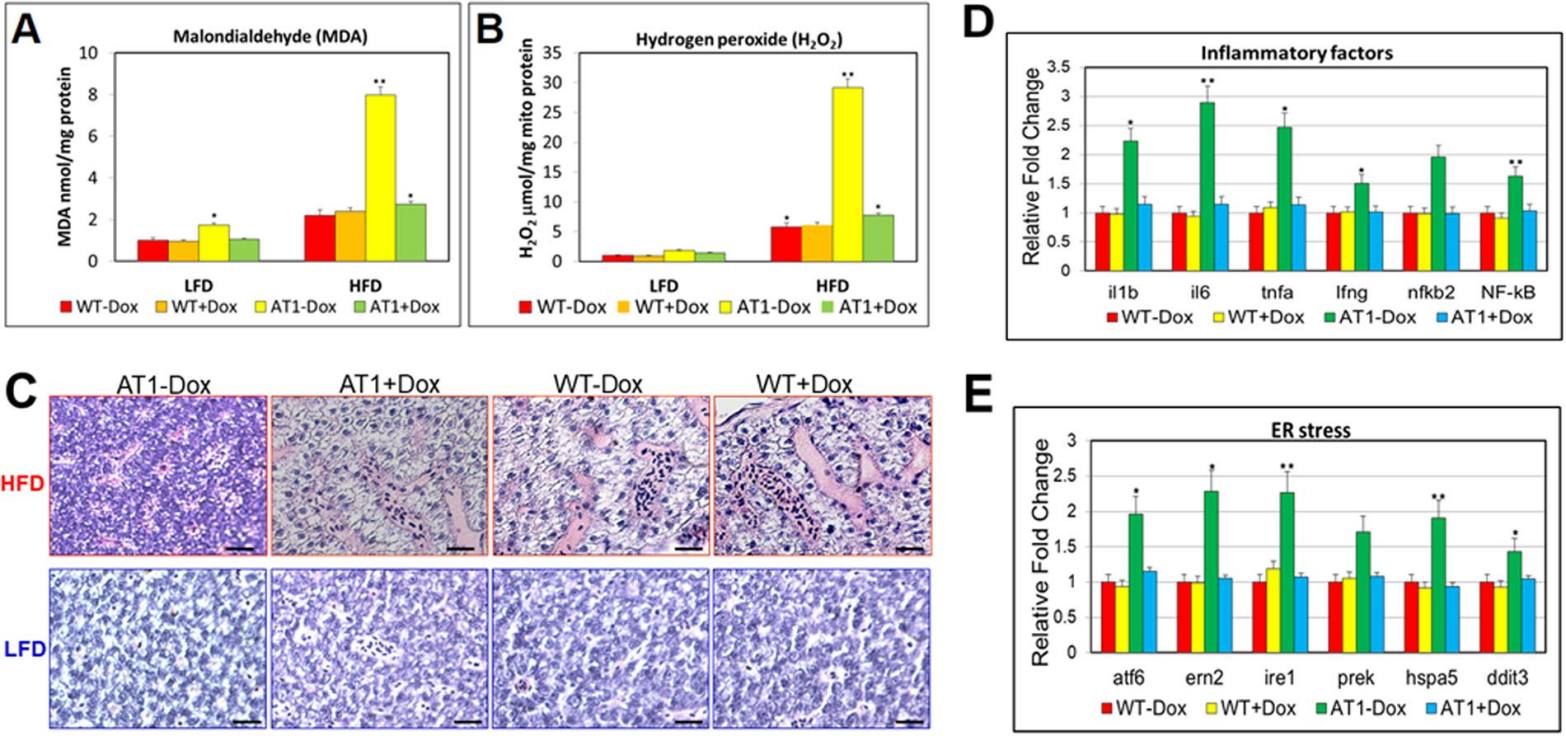
ATF4 overexpression sensitizes zebrafish to oxidative stress induced by a HFD and induces NASH. (**A**) Levels of hepatic MDA, and (**B**) H_2_O_2_ in hepatic mitochondria was compared between AT1 ± Dox and WT ± Dox adults fed a HFD or LFD for 4 weeks. (**C**) Representative histological analysis of livers from the AT1 ± Dox and WT ± Dox adults that were fed a HFD or LFD for 4 weeks (400X magnification, scale bars: 10 μm). (**D**) Molecular analysis of AT1-Dox NASH-like livers revealed the upregulation of inflammatory genes, *il-1b*, *il-6*, *tnf-α*, *ifn-γ*, *nfkb2*, and *NF-kB*. (**E**) Molecular analysis of AT1-Dox NASH-like livers revealed the upregulation of ER stress markers, atf6, *ern2*, *ire1*, *prek*, *hspa5*, *grp78*, and *ddit3*. The biochemical analysis of oxidative stress were performed in triplicate in (**A**,**B**) and prepared from average 2–3 eight months male–female fish pairs per groups (WT-Dox, WT + Dox, AT1-Dox, AT1 + Dox). The qRT-PCRs were performed in triplicate in (**D**,**E**). Expression analysis of the selected genes using cDNA prepared from average 2–3 eight months male–female fish pairs per groups (WT-Dox, WT + Dox, AT1-Dox, AT1 + Dox). Levels of mRNA were normalized to β-actin and expressed as fold of values in the WT-Dox control. The asterisk represents statistically significant differences; *p < 0.01, and **p < 0.005.

To investigate whether ATF4 is also important for regulating oxidative stress in the liver of AT1 − Dox, AT1 ± Dox and WT ± Dox were fed a HFD or low fat diet (LFD) for 4 weeks, which previously showed that ATF4 deficiency may ameliorate HFD-induced oxidative stress in the mouse liver^17^. We discovered that HFD-induced oxidative stress significantly increased the quantity of hepatic MDA and release of H_2_O_2_ in ATs − Dox; however, no or less effect of oxidative stress was observed in the liver of AT1 ± Dox and WT ± Dox fed with the LFD (Fig. 5A and B), suggesting that ATF4 overexpression can significantly enhance HFD-induced oxidative stress in AT1 − Dox livers. Furthermore, a HFD-induced NASH-like phenotype was observed in the AT1–Dox group, but not in the AT1 + Dox and WT ± Dox groups, as demonstrated by H&E-stained sections (Fig. 5C). Furthermore, the molecular analysis of AT1 − Dox NASH-like livers revealed upregulation of related genes involved in NASH development. As expected, AT1 − Dox adults exhibited increased expression of the inflammatory genes *il-1b*, *il-6*, *tnf-α*, *ifn*-γ, *nfkb2*, and *nf-kb* compared with the AT1 + Dox and WT ± Dox adults (Fig. 5D). In addition, AT1 − Dox adults exhibited upregulation of ER stress markers *atf6*, *ern2*, *ire1*, *prek*, *hspa5*, and *ddit3* (Fig. 5E). These results indicate that AT1 − Dox adults developed NASH because of liver oxidative stress and subsequent activation of inflammatory and ER stress pathways.

### ATF4 overexpression enhances early onset of adipocyte hyperplasia in zebrafish

**Figure 6:**
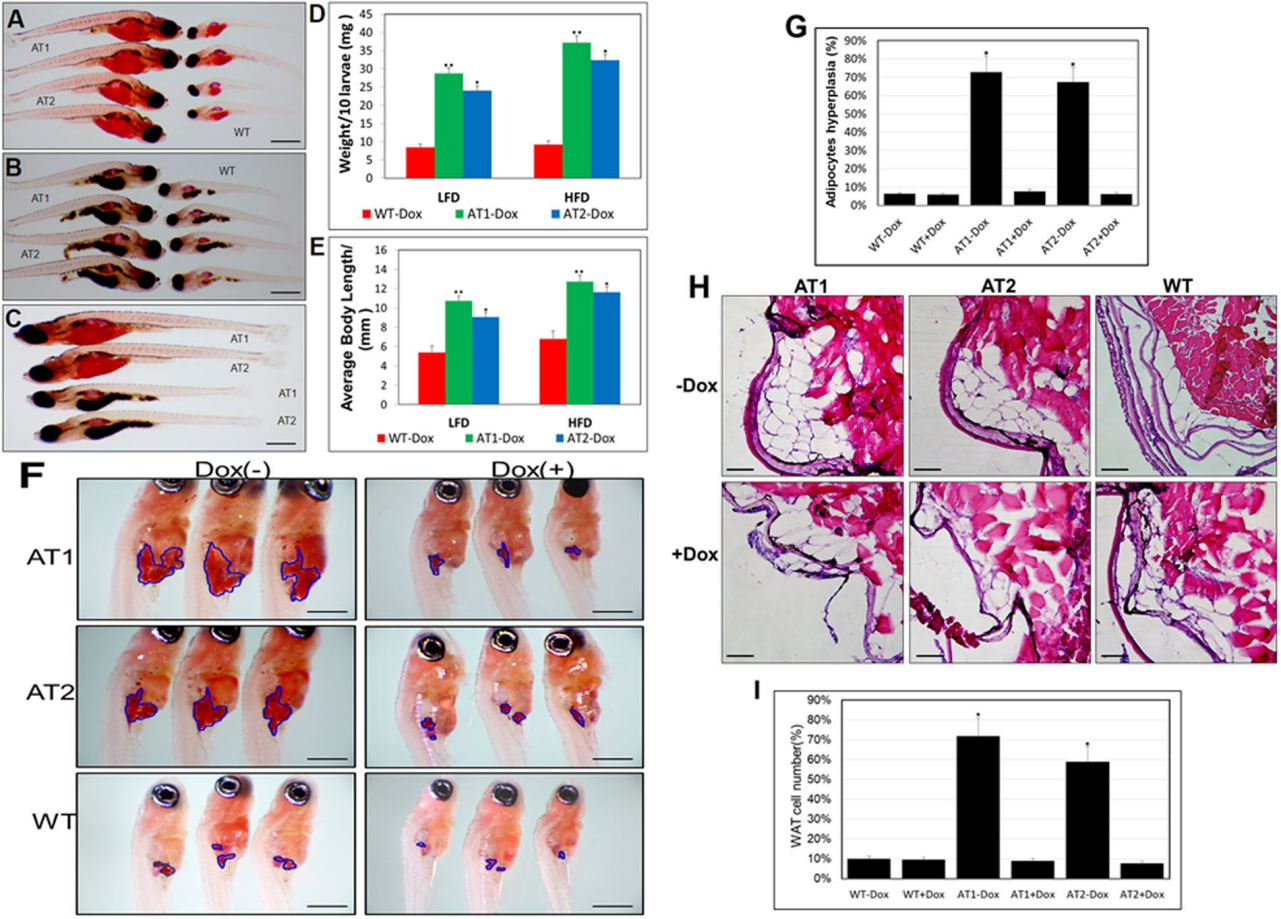
ATF4 overexpression results in enhancement of ATs larval growth and adipocyte formation (**A**) Length comparison of ATs − Dox and WT-Dox larvae fed a HFD for two weeks. (32X magnification, scale bars: 200 μm). (**B**) Length comparison of ATs − Dox and WT-Dox larvae fed a low-fat diet (LFD) for two weeks (32X magnification, scale bars: 200 μm). (**C**) Length comparison of ATs − Dox larvae fed a HFD or LFD for two weeks (32X magnification, scale bars: 200 μm). (**D**) Average body weights of ATs − Dox and WT − Dox larvae fed a LFD or HFD (n = 40–50 for each groups). (**E**) Average body lengths of ATs − Dox and WT − Dox larvae fed a LFD or HFD (n = 40–50 for each groups). (**F**) ORO-stained visceral adipocytes in ATs − Dox compared to control zebrafish at 24 dpf (40X magnification, scale bars: 200 μm). Visceral adipocytes are circled. (**G**) The percentage of zebrafish larvae containing hyperplasia of visceral adipocytes (n = 40–50 for each groups). (**H**) ATF4 overexpression leads to adipocyte hyperplasia. H&E stain of the abdominal white adipose tissue (WAT) sections from 24 dpf ATs ± Dox and WT ± Dox fed a HFD for 2 weeks (400X magnification, scale bars: 10 μm). (**I**) The percentage of cell number in intra-abdominal fat pads of 24 dpf ATs ± Dox and WT ± Dox fed a HFD for 2 weeks (n = 10–15 for each groups). The asterisk represents statistically significant differences; *p < 0.01, and **p < 0.005.

To evaluate the potential importance of ATF4 overexpression in early zebrafish adipogenesis, we analysed AT larvae and WT siblings of the same stage fed with a LFD or HFD. While both were greater than that of the WT, body weight and length were markedly increased in ATs − Dox fish fed with a HFD compared to those in fish fed a LFD during larval development (Fig. 6A–E). In addition, we compared the number of ORO-stained adipocytes in ATs − Dox compared to that in control zebrafish at 24 dpf. We detected an increased cell mass of visceral adipocytes (Fig. 6F) and an increased percentage of zebrafish larvae containing hyperplasia of visceral adipocytes compared to that in control zebrafish (Fig. 6G). The differences in hyperplasia of visceral adipocytes are apparent in H&E–stained sections, which showed that ATs − Dox fish have a significant fat mass compared with the paucity of such deposition in WT fish (Fig. 6H). The level of hyperplasia of visceral adipocytes were further quantified by measuring adipocyte number (cells/field of view) in the hematoxylin and eosin (H&E)–stained sections of abdominal fat pads excised from ATs ± Dox and W ± Dox fish (Fig. 6I). Thus, ATF4 overexpression resulted in hyperplasia of visceral adipocytes in zebrafish larvae implicating an effect of ATF4 expression on postembryonic growth and larval adipocyte formation of zebrafish.

### Adult ATs are plump and have increased WAT mass

**Figure 7:**
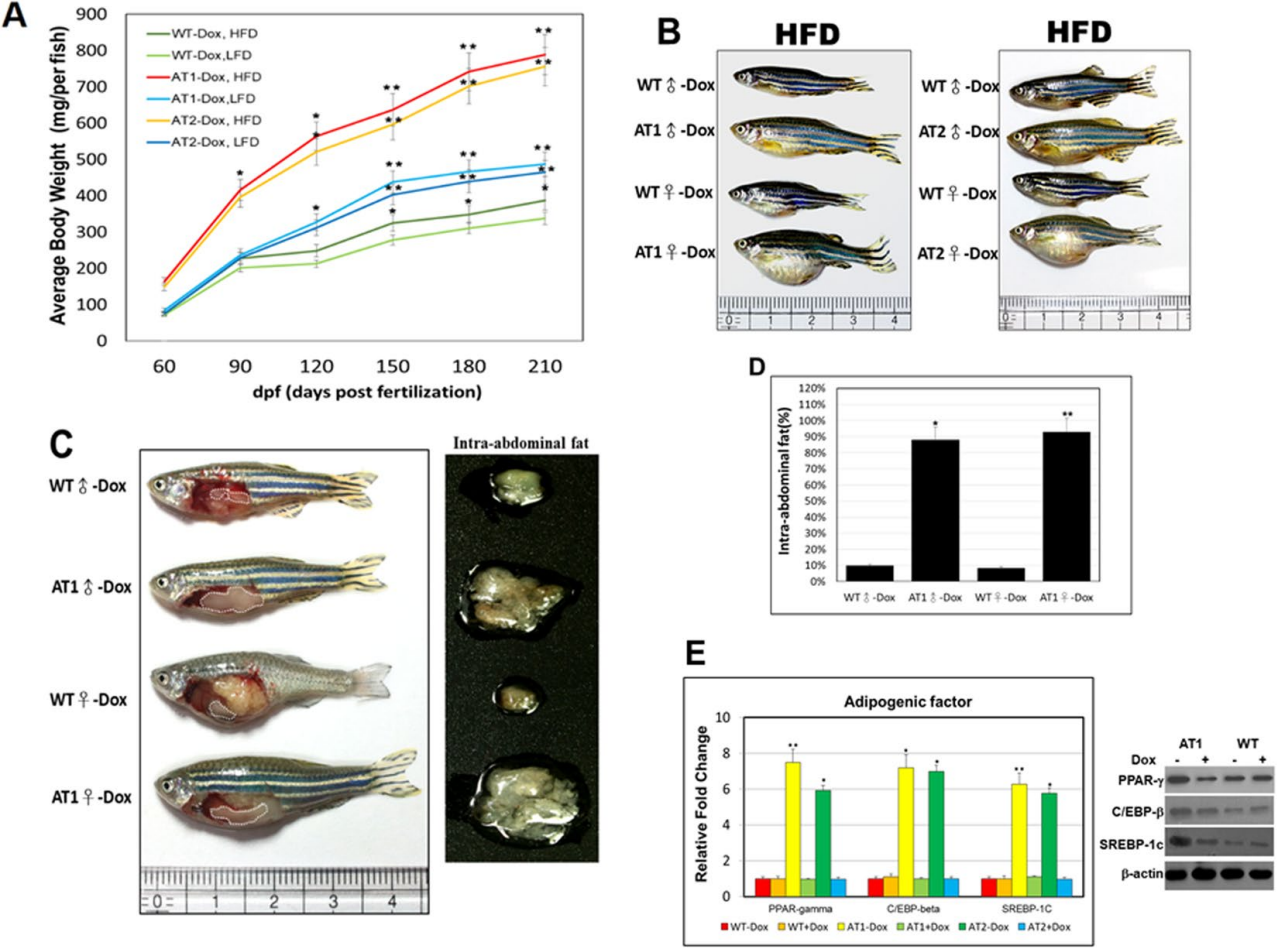
ATF4 overexpression induces weight gain and increases fat mass. (**A**) ATs were fed either a LFD or HFD for the indicated time periods. Serial body weight of each zebrafish was monitored monthly (n = 20–25). *p < 0.01, and **p < 0.005. (**B**) Representative images of male and female ATs − Dox and WT-Dox fed either a HFD for 5 months. (**C**) ATF4 overexpression leads to greater intra-abdominal fat (left), and the increased intra-abdominal fat pad size is shown (right). (**D**) Nuclear magnetic resonance intra-abdominal fat analyses of 10-month-old HFD–fed WT-Dox and AT1-Dox. Examined fish was prepared from 5–8 four months male–female fish pairs of each indicated groups fed either a HFD for 6 months. The asterisk represents statistically significant differences; *p < 0.01, and **p < 0.005. (**E**) Expression of adipogenic genes and proteins. *PPAR-γ*, *C/EBP-β*, and *SREBP-1c* mRNA (left, qRT-PC), and proteins (right, western blot). The qRT-PCRs were performed in triplicate in (E). Expression analysis of the selected genes using cDNA prepared from average 2–3 eight months male–female fish pairs per groups. Levels of mRNA were normalized to β-actin and expressed as fold of values in the WT-Dox control. The asterisk represents statistically significant differences; *p < 0.01, and **p < 0.005.

To determine whether overexpression of AT4 might have functional relevance in adipose tissues, we examined the phenotype of the adipose tissues of adult ATs and found that they had a hypertrophic response to obesity or overweightness. During the 4 months of feeding, we found that ATs − Dox zebrafish had a significantly sharp response to the HFD. Consistent with the growth curve, the body weight in ATs − Dox adults was dramatically increased within the 4 months (Fig. 7A). ATs − Dox adults were plump and larger compared to WT adults fed a HFD (Fig. 7B). This intensification to diet-induced weight gain was accompanied by a marked increase in fat accumulation as determined by nuclear magnetic resonance analyses as well as by gross observation and weights of fat depot explants (Fig. 7C,D). We also examined whether ATF4 modulates adipogenesis in the WAT of ATs accompanied primarily with lipid accumulation. The lipid accumulation was accompanied by an increased expression of adipogenic transcriptional factors, including PPAR-γ, C/EBP-β, and SREBP-1c (Fig. 7E). These results suggest that overexpression of ATF4 accelerates adipocyte differentiation in ATs, resulting from upregulated adipogenic transcriptional factors involved in adipogenesis.

## Discussion

Zebrafish lipids are normally stored as TGs^34^, and the main storage sites include visceral, intramuscular, and subcutaneous lipid depots, however their storage is poor in blood vessels^35,36^ and the liver^37–39^. The neutral lipid stain ORO was recently proposed for monitoring endotrophic lipid consumption during zebrafish embryonic and larval stages^35,40,41^. We observed minimal ORO staining in the swim bladder, heart, head, and vasculature of control larvae (ATs + Dox and WT). ATF4 overexpression resulted in strong ORO staining of the liver and vasculature structures. One explanation for the increase in ORO staining in the ATs − Dox larvae may be the enhancement of lipogenesis. Although ATF4 is a key transcription factor in regulating lipids biosynthesis and is a contributing factor for hypertriglyceridemia^16^, the detailed mechanism of its action remains unclear. In agreement with our data, studies using *ATF4* knock-out mice demonstrated that ATF4 deficiency decreases the synthesis of fatty acids by down-regulating the expression of lipogenic genes and increases lipolysis and β-oxidation^13^. It has also been demonstrated that ATF4 plays an essential role in regulating SREBP-1c expression in 3T3-L1 adipocytes^42^, and ATF4 would stimulate lipogenesis and instigate hepatic steatosis in mice^16^. Overall, our data support the view that the increase in ORO staining in the blood vessels of ATs − Dox zebrafish was due to an increase in the concentration of plasma lipid. Our results suggest parallel lipid metabolic pathways for ATF4 expression *in vivo*, and strong similarities between the lipid metabolisms of ATF4-mediated regulation in mammals and zebrafish.

It has been shown that ATF4 functions as a transcriptional repressor in many cases^43^. Importantly, Li *et al*. and Wang *et al*. indicated that ATF4 may also function as a transcriptional activator in hepatocytes^14,17^. They indicated that ATF4 is a stress transcription factor that is expressed in the liver in response to a HFD or HCD (high-carbohydrate diet), and may be able to alleviate some of the HFD-induced oxidative stress^17^ or HCD-induced hepatic steatosis^14^. In addition, It has been found that overexpression of ATF4 up-regulates SCD1 (stearoyl-CoA desaturase 1) and SREBP-1c expression *in vivo*, and increases TG accumulation in mouse livers^14,16^. Induction of ATF4 was also observed in a number of human disease states accompanied by oxidative stress including atherosclerosis, allergic contact dermatitis, and sporadic Alzheimer’s disease^44–46^. Correspondingly, our *in vivo* observations indicate that HFD feeding results in significantly increased oxidative stress in the liver of ATs − Dox zebrafish. Moreover, the levels of MDA and H_2_O_2_ release were also significantly increased in response to ATF4 overexpression. Our results suggest that ATF4 is involved in the regulation of HFD-induced oxidative stress.

The mechanism by which UPR activation causes lipid accumulation in hepatocytes is not known. We found that ATs − Dox-induced steatosis is accompanied by induction of UPR target genes, which is similar to findings of other studies. Vacaru *et al*. demonstrated that zebrafish liver steatosis is associated with markers of UPR activation and robust UPR induction can cause steatosis^47^. Previous studies have demonstrated that treatment with the ER stress inducer tunicamycin leads to hepatic steatosis in zebrafish larvae^48,49^. Furthermore, we provided evidence that ATF4 expression in the liver is responsible for the development of hepatic steatosis and even steatohepatitis, as further combinational effects on HFD-induced oxidative stress^50^.

Several studies have revealed that ATF4 also plays an important role in adipogenesis^18,19,42^. Recently, results from two research groups have suggested that ATF4 is a positive regulator of adipocyte differentiation^18,19^. We demonstrated that ATF4 overexpression is sensitive to HFD-induced adipocyte hyperplasia, which, at least partially, accounts for the weight gain or obesity in ATs − Dox. We showed that the ATs can be used as a model for early-onset weight gain after embryonic Dox exposure. ATs − Dox zebrafish grew fast during the larval stages (10–21 dpf), resulting in an increased body weight and length at larval stages. Possibly, the increased body weight could be the result of a “head-start” in growth caused by early-life lipid accumulation, which may be associated with lipid metabolic impairment and an energy consumption deficient state, thereby affecting postembryonic growth of zebrafish^51,52^.

Mice lacking ATF4 are lean and resist diet-induced obesity, although they have adipose tissue^7,53,54^. Based upon appearance and examination of adipose depots, the larval ATs − Dox had modestly increased fat mass and ATs − Dox young adults had significantly increased fat mass compared with the controls. Obviously, this effect substantially increased in adults, indicating that ATs − Dox were sensitive to age-associated weight gain. In addition, our results also suggest that the increased fat mass in ATs − Dox was caused by an increase in cell number (hyperplasia of adipocytes). We hypothesized that increase of adipose volume in ATs − Dox results from increased adipogenesis. Consistent with this possibility, recently, Cohen *et al*. demonstrated a functional role for ATF4 in adult stem cell adipocyte differentiation, suggesting that C/EBP-β controls distinct gene expression programs in the 3T3-L1 cell line^18^ and adipose tissue^19^. Results from animal model research suggests that ATF4 is functionally associated with obesity, glucose homeostasis, fat storage, and energy expenditure during fly and mouse development^13,53,54^. Our results suggest that some of these ATF4 functional attributes may be conserved in adipogenesis, which has been shown to be closely related to the general development and size of zebrafish.

In conclusion, our results showed that chronic ATF4 expression could increase lipogenesis with an impact on oxidative stress and adipogenesis, which in turn would drive fat accumulation and enhanced weight gain in zebrafish. The proposed juvenile AT models showed different pathological states such as hepatic steatosis and, more variably, steatohepatitis and hyperlipidaemia. Aged ATs with transmissible NASH-like and obesity phenotypes (different levels of severe hepatic steatosis and fat mass) are promising models for studying human metabolic diseases such as hypertriglyceridemia.

## Methods

### Plasmid constructs and generation of transgenic zebrafish

*ATF4*-V5 and 2A-e*GFP* fragments were amplified by polymerase chain reaction (PCR). The *ATF4*-V5 fragment was amplified using an orthologous zebrafish *ATF4* gene sequence (GenBank Accession No. BC067714) forward primer and an *ATF4* reverse primer, each containing a V5 sequence at the 3′ end. The 2A-*mCherry* fragment was amplified using a mCherry forward primer and an mCherry reverse primer, each containing the normal 2 A peptide sequence at the 5′ end^55^. We then constructed the pβ-Act-Tet^off^-ATF4–2A-mCherry plasmid from the pLF2.8-Tet^off^-CB1R-2A-eGFP^37^ plasmid by cloning in the 2A-*mCherry* and the *ATF4*-V5 [Fig. 1(A)].

We used the pβ-Act-Tet^off^-ATF4–2A-mCherry transgenic construct to generate ATF4 transgenic lines (ATs). The ATs, [Tg (−2.5β-Act:Tet^off^-ATF4-2A-mCherry)], showed that conditional global expression of *mCherry* and zebrafish *ATF4* genes is driven by the promoter of the *β-actin1* gene (GenBank Accession No. NW_001878018). For experimental assays of zebrafish growth, zebrafish larvae were fed twice a day, starting at the 5 dpf, with either low fat diet (LFD; AZO, Taikong CORP.,Taiwan) or high fat diet (HFD; 10% crude fat added to AZOO LFD diet) for 7 days. The zebrafish were maintained at our own facility in a controlled environment under a 14/10 h light-dark cycle at 28 °C. All zebrafish experiments were performed in accordance with relevant guidelines and regulations of the National Taiwan Ocean University (NTOU). This study was approved by the Animal Ethics Committee of the NTOU (permit number NTOU-104042 and 104016).

### Doxycycline (Dox) treatment

Homozygotic AT embryos were kept in water until 24 hours post fertilization (hpf). AT embryos were then treated with Dox (10 μg/ml). Three-month-old AT and wild-type (WT) adult fish were selected to undergo Dox suppression treatment. Ten fish from each group were raised in tanks and treated with 25 μg/ml Dox. The water in the tanks was replaced daily. After suppression, the mCherry fluorescence signal and the morphological changes were observed under an MZ16 FA fluorescence stereomicroscope (Leica, Wetzlar, Germany).

### Western blot analysis

Total protein was extracted from isolated zebrafish samples, using a lysis buffer (0.5% NP-40, 20 mM Tris-HCl, pH 8.0, 100 mM NaCl, and 1 mM EDTA). Protein concentration was determined using a Bio-Rad protein assay kit (Bio-Rad, Hercules, CA). Hepatic protein extract (20–50 μg) was separated by 12% sodium dodecyl sulphate–polyacrylamide electrophoresis and transferred to a polyvinylidene difluoride membrane (Bio-Rad). The membrane was blocked in 5% non-fat dried milk in phosphate-buffered saline 0.05% (v/v) and Tween 20 (PBST; pH 7.4) (Sigma-Aldrich, St. Louis, MO) and probed with antibodies that had been diluted at 1:500–1000 in a 0.3% solution of bovine serum albumin in PBST. Anti-V5 antibody was purchased from Invitrogen. Anti-PPARγ, anti-C/EBP-β, and anti- SREBP-1c antibodies were purchased from Santa Cruz. β-actin served as the loading control and detected using anti-β-actin antibody (Sigma-Aldrich) diluted at 1:1000 in PBST.

### Lipid staining of zebrafish larvae

Sliced liver samples or larvae were washed three times with PBS and then fixed with 10% formalin in a phosphate buffer for 1 h at room temperature. The samples were again washed with PBS, stained with a filtered Oil Red O (ORO) (Sigma-Aldrich) stock solution (0.5 g ORO in 100 ml isopropyl alcohol) for 15 min at room temperature, and then washed twice with H_2_O for 15 min. For ORO whole-mount analysis, whole larvae were fixed with 4% paraformaldehyde, washed with PBS, infiltrated with a graded series of propylene glycol baths, and stained with 0.5% ORO in 100% propylene glycol overnight. Stained larvae were washed with decreasing concentrations of propylene glycol, followed by several rinses with PBS, and transferred to an 80% glycerol bath.

### Measurement of lipids, malondialdehyde (MDA), and hydrogen peroxide (H_2_O_2_) in zebrafish livers

Biochemical analyses of zebrafish liver lipids were performed as previously described^37^. Hepatic MDA and H_2_O_2_ levels were determined using commercial kits (Wako, Chuo-ku, and Osaka, Japan).

### Quantitative real-time reverse transcriptase PCR (qRT-PCR)

DNase-treated hepatic total RNA (2 μg) was reverse-transcribed into cDNA, using an iScript cDNA Synthesis Kit (Bio-Rad, Hercules, CA) following the manufacturer’s protocol. For qRT-PCR, serial dilutions of cDNA were prepared in a TE buffer (10 mM Tris-HCl, 1 mM EDTA, pH 8.0). The reactions were carried out using a 2 × LightCycler 480 SYBR Green I Master system. The GenBank accession numbers for the selected genes and primer sequences were previously reported^37–39,56^. Each sample analysis was carried out in triplicate. β-actin was evaluated for use as an internal control for qRT-PCR.

### Statistics

Experimental values are expressed as the mean ± SEM. A statistical analysis was performed by the Student’s t test for unpaired data, with *p* < 0.01 considered to indicate statistical significance.

## Electronic supplementary material

Supplementary Information
