# Supplementary material for: ATF4 overexpression induces early onset of hyperlipidaemia and hepatic steatosis and enhances adipogenesis in zebrafish
Source: Sci Rep. 2017 Nov 27;7:16362. doi: 10.1038/s41598-017-16587-9 (PMC5703967; doi:10.1038/s41598-017-16587-9)
Supplement: Supplementary file 1 — Supplementary Information [file 41598_2017_16587_MOESM1_ESM.doc]

**ATF4 overexpression induces early onset of hyperlipidaemia and**

**hepatic steatosis and enhances adipogenesis in zebrafish**

Kun-Yun Yeh, Chi-Yu Lai, Chiu-Ya Lin, Chia-Chun Hsu, Chung-Ping Lo and Guor Mour Her

**Supplementary Fig S1**

**
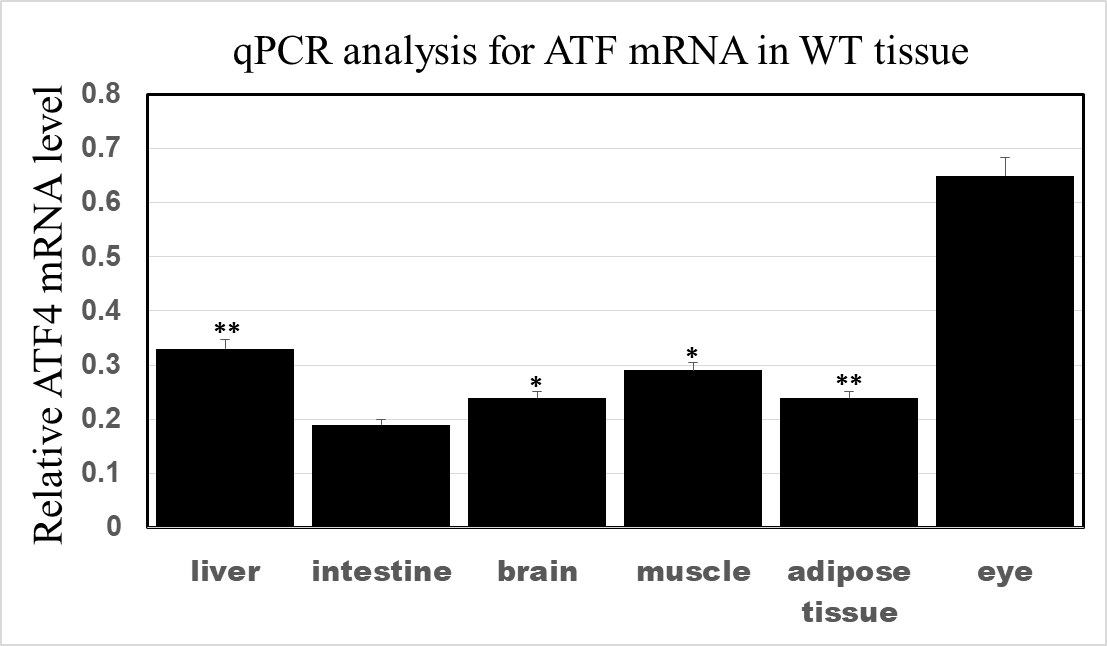
**

**Supplementary Fig. S1.** RT-qPCR analysis of ATF4 mRNA expression in various tissues of adult zebrafish, including the liver, intestine, brain, muscle, adipose tissue, and eye. The qRT-PCRs were performed in triplicate. Expression analysis of the selected genes using cDNA prepared from average 2-3 four months male–female fish pairs. Levels of mRNA were normalized to β-actin. The asterisk represents statistically significant differences; * p < 0 .01, and ** p < 0.005.
